# A comparative analysis of the role of containment policies, vaccination strategies and virus variants in the COVID-19 pandemic across nine European countries

**DOI:** 10.1038/s41598-025-10132-9

**Published:** 2025-08-08

**Authors:** Simone Rancati, Giovanna Nicora, Enea Parimbelli, Marco Salemi, Riccardo Bellazzi, Daniele Pala

**Affiliations:** 1 Department of Computer, Electrical and Biomedical Engineering, University of Pavia, Pavia, Italy; 2 Emerging Pathogens Institute, University of Florida, Gainesville, FL USA; 3 Department of Pathology, Immunology and Laboratory Medicine, College of Medicine, University of Florida, Gainesville, FL USA; 4 Department of Management, Information and Production Engineering, University of Bergamo, Bergamo, Italy

**Keywords:** COVID-19 containment policies, Epidemiological modeling, Multilevel vector autoregression (mlVAR), Policy stringency index, Vaccination impact, SARS-CoV-2 variants, Health policy, Public health, Epidemiology, Biomedical engineering, Infectious diseases

## Abstract

The COVID-19 pandemic disrupted public health, economies, and societies worldwide, with lasting effects still visible today. Containment policies were implemented to reduce viral transmission, but their effectiveness varied across geographic regions. Understanding the interplay between policies, epidemiological variables, vaccines, and variants is crucial for optimizing future epidemic responses. This study applies a vector autoregressive mixed-effect model to data from nine European countries over the entire pandemic duration, using weekly data to provide both global and country-specific analyses. Policy stringency was assessed using the Oxford Covid-19 Government Response Tracker. Our findings reveal that the relationship between epidemiological indicators and policy stringency evolved over time. Initially, hospitalizations were the primary driver of containment measures, but as vaccinations increased and new variants emerged, new case numbers became an important factor too. While all vaccine doses contributed to reducing cases and hospitalizations, only the second dose was associated with policy stringency. No significant association was found between virus variants and policy measures. Despite small country-specific differences, these insights highlight the complex interdependencies that shaped pandemic management. This framework can support future public health decision-making by balancing epidemiological trends with tailored policy responses at both national and international levels.

**Supplementary Information:**

The online version contains supplementary material available at 10.1038/s41598-025-10132-9.

## Introduction

The COVID-19 pandemic has been one of the most significant global events of the 21st century so far, as it caused a series of disruptions in public health, economics and politics that affected the entire planet with effects still lingering years later. The pandemic started in December 2019 and was declared finished in May 2023^1^. In this period, COVID-19 caused 760.79 million registered infections and 6.90 million deaths in the World, with an average of 71,916 cases and 1498 deaths every week^2,3^. It was caused by the Sars-CoV-2 virus, a new pathogen belonging to the Coronavirus family that possesses the ability to spread very quickly and lead, in the initial phases of the pandemic, to the rapid rise of hospitalization and mortality rates. Although coronaviruses possess an nsp14-mediated proofreading activity that lowers their intrinsic mutation rate relative to many RNA viruses, the massive scale of global transmission and episodic acceleration in certain hosts have allowed SARS-CoV-2 to accumulate genetic changes at an effective rate of ~ 10^−3^ substitutions per site per year—sufficient to fuel the continual emergence of novel variants^4,5^ such as Alpha (B.1.1.7), Beta (B.1.351), Gamma (P.1), Delta (B.1.617.2), or the currently circulating Omicron (B.1.1.529) and their (sub)lineages like XBB.1.5. This situation represented a public health emergency, with the first few outbreaks quickly escalating to the overwhelming of most healthcare systems, as countries did not have the hospital beds and resources to treat an unprecedented number of people with cardio-respiratory complications^6^. In order to prevent further spreading of the disease, national and local administrations started emanating containment policies that had the aim of reducing the probability of contacts within the population, for example mandating social distance and use of face masks, prohibiting leisure travel, canceling public events, and closing food services. These policies had inevitable socioeconomic side effects and a tremendous impact also on the populations’ mental health^7^. This was probably partly due to the fact that the initial response to the spreading of the virus was unorganized, as the novelty of the pathogen and the unprecedented emergency situation made it impossible to create data-driven responses, therefore the public health interventions were not optimized to face the pandemic. As the pandemic progressed, numerous theories about the mechanisms that lead to high mortality and morbidity were made, however it took time to collect and analyze the necessary data to confirm them. Knowledge of these mechanisms could have allowed for more targeted containment policies, tailored to minimize both the public health damage and the socioeconomic consequences of the restrictions at the same time. Several studies aimed at optimizing containment policies, but the validity of the results was constantly undermined by the fast changes in the epidemiological situation, continuously occurring with the introduction of new variables such as the vaccination rates and the new variants of the virus.

This study presents an overall description of the various phases of the pandemic in several European countries, with a particular focus on the reaction of those countries in terms of containment policies and their association with the epidemiological outcomes. Our aim is to provide a global overview of how the public health policy makers reacted to the spreading of the virus, both at the initial stage of the pandemic and in the subsequent phases, characterized by the introduction of large vaccination campaigns and the birth of new, often more infectious, variants. We focused on nine countries that were selected based on the completeness and availability of their data: Cyprus, Estonia, France, Greece, the Netherlands, Latvia, Ireland, Slovenia and Spain. We used a multilevel vector auto-regressive mixed-effects model (mlVAR), considering each country as a member of a population and including them as random effects in the model, in order to take into account both the general relationships among the different variables in the entire population and the local effects for each individual country. This approach allows modeling how the complex interactions among different aspects of the pandemic evolved in time, using epidemiological and socio-political data that we collected from a variety of diverse sources (OxCGRT for policy stringency, ECDC for epidemiological variables and GISAID for data related to the virus’ variants), and reported with a weekly granularity for the three years of the pandemic.

### Previous work

This work has been conceived as the continuation of a research that started during the first year of the pandemic as part of the PERISCOPE project. The PERISCOPE project, funded by the European Commission, created a multi-disciplinary consortium made of both academic and industrial partners to undertake research on the COVID-19 pandemic in order to help policy makers manage the pandemic and be prepared for the new ones. One of the main aims of PERISCOPE was the creation of a European COVID-19 Atlas^8^, in which data from a variety of different sources was integrated and summarized to create visualization tools and statistics about the epidemiological, economic, and socio-political indicators during the pandemic. Other scopes of the project included the development of descriptive and predictive models to assess the relationships between different aspects of the pandemic impact in order to better understand its epidemiological and social dynamics, including the interplay between containment policies and epidemiological variables. In the context of this project, we performed a study that was published in 2023 in the journal Scientific Reports^9^. In this paper, we used a multilevel vector autoregressive model (mlVAR) to study the two-way relationship between non-pharmaceutical interventions and epidemiological manifestations of COVID-19 in nine selected countries in Europe. The results of this study provided important information on the complex interplay between health policies, assessed using the CoronaNet database^9^, and epidemiological surveillance, but also showed how the effectiveness of specific policies was rather country-specific, although with some common ground given by the fact that policies aimed at increasing health monitoring and resources were the most effective in all countries. The study, however, was performed only on 2020 data, as data about the vaccination rates and the advent of new variants were not yet available in sufficient quantity to assess their mutual influence with the governments’ policies and the consequent impact on infection, hospitalization and death rates of the disease. Other studies concerning the link between health policies and epidemiological outcomes have been conducted in the last years, Antonini et al.^10^ published an editorial about the shift of the pandemic towards endemicity focusing on how vaccinations changed the COVID-related policies during the pandemic. Deb et al.^11^ discussed the link between increasing vaccinations and economic activity, and some other studies discussed the link between policy stringency and some aspects of the pandemic, such as the one from Teddy et al.^12^ that analyzed the effect of policy stringency on health services disruption in 10 countries (non-European). All these studies present valuable contributions to the literature, but they all tend to focus on one or a few particular aspects of the pandemic or on a specific geographic area, while our previously published study, to the best of our knowledge, was the only one to provide an integrative overview of the interplay between various aspects of the pandemic period in Europe. The novel study described in this paper presents an extension of that analysis that includes newer data from the years 2021 and 2022, when the introduction of vaccinations and new variants forced the policy makers to continuously change their strategies. With these new results, we are able to provide better insights both on the mechanisms that influenced the strengthening or release of containment measures, and vice versa on those that led from public health policies to a change in the epidemiological situation of the pandemic, taking into account all the different variables that characterized each phase.

## Methods

### Data sources and preprocessing

Three main data sources were used for our analysis: OxCGRT^13^, ECDC^14^ and GISAID^15^. Data preprocessing was performed using both RStudio and PyCharm. The first preprocessing step, which is common across all datasets, involved applying a filter to retain only the data generated between January 2020 and December 2022. Additionally, each dataset was split based on the country of interest: Spain, France, the Netherlands, Latvia, Slovenia, Greece, Ireland, Cyprus, and Estonia.

#### Oxford COVID-19 government response tracker

**Fig. 1:**
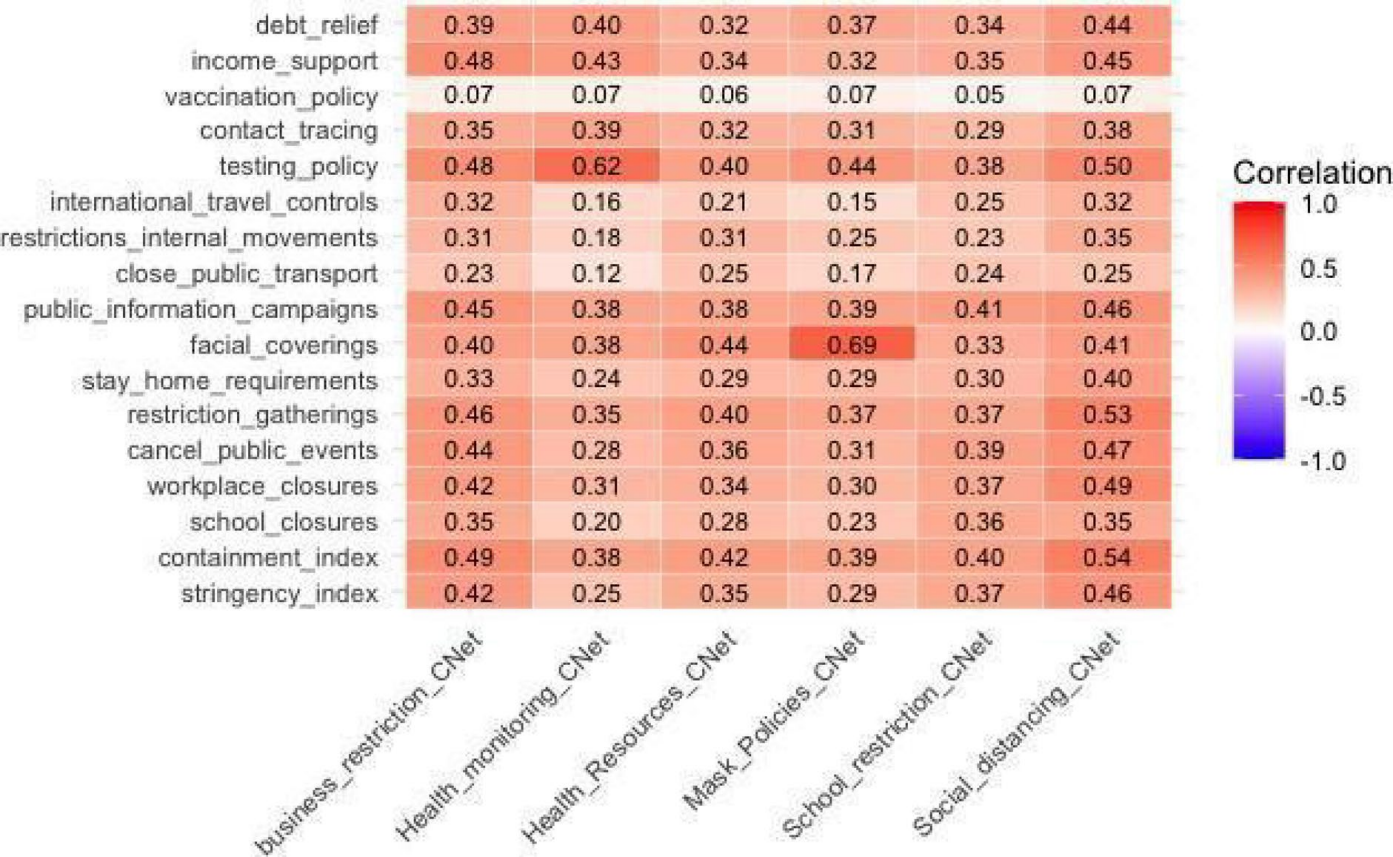
Global Correlation Matrix of OxCGRT Indicators and CoronaNet Policy-Intensity Indices (PIIs) for 2020. This heatmap displays the Pearson correlation coefficients between various OxCGRT metrics, including the Stringency Index, and the six Policy Intensity Indices (PIIs) developed in the CoronaNet Project (CNet) in 2020. Notably, 2020 marks the last year in which the CNet indices were updated. The Stringency Index shows moderate correlations with all PIIs, particularly with the Business Restrictions Index (0.42), Social Distancing Index (0.46), Health Monitoring Index (0.25), Mask Policies Index (0.29) and School Restriction Index (0.37).

The Oxford COVID-19 Government Response Tracker (OxCGRT)^13^ is a comprehensive project aimed at documenting and analyzing government responses to the COVID-19 pandemic. Over the years 2020, 2021, and 2022, the dataset captured real-time variations in policy stringency i.e., the strictness of governmental actions (policies), including public health measures, economic policies, and mobility restrictions, providing valuable insights into the global response to COVID-19. One of the primary tools developed by the project is the Stringency Index (SI).This index is calculated starting from nine key metrics: school closures, workplace closures, cancellation of public events, restrictions on public gatherings, public transport closures, stay-at-home requirements, public information campaigns, restrictions on internal movements, and international travel limitations. These metrics collectively reflect the government action implemented by various countries to reduce COVID-19 infection rates. The SI is computed as the simple, unweighted average score of these nine metrics, each ranging from 0 to 100, with a higher score indicating a stricter government response i.e., higher policy stringency. When policies differ at the subnational level, the index reflects the strictest region. It is important to note that the SI measures the strictness of policies starting from January 2020, but does not assess their effectiveness or appropriateness, so a higher score does not necessarily imply a better response. In addition to the SI, other measures, such as the Policy Intensity Indices (PIIs)^9^, have been developed, notably by Kubinec in the CoronaNet Project (CNet) and used in prior research, including our previous study^9^. The choice to use the SI from OxCGRT instead of the PIIs in this analysis is driven by the availability of the SI for the years 2022 and 2023, unlike the PIIs. Furthermore, the SI consolidates all relevant metrics into a single measure, offering ease of interpretation when analyzing results, whereas the PIIs are characterized by six distinct indices (Social Distancing, School Restrictions, Business Restrictions, Health Monitoring, Health Resources, and Mask Policies) without a single combined index. An exploratory analysis was conducted to assess the relationship between OxCGRT indices, including the SI, and the PIIs. Figure 1 presents the Pearson correlation between the OxCGRT indices and the PIIs, with a particular focus on the correlation between the Stringency Index (SI) and all PIIs. The results show the diffuse presence of moderate correlations between the different variables used to define the two indexes and indicates that the SI serves as an effective summary measure, capturing the overall trends represented by the individual PIIs.

#### European centre for disease prevention and control

**Fig. 2:**
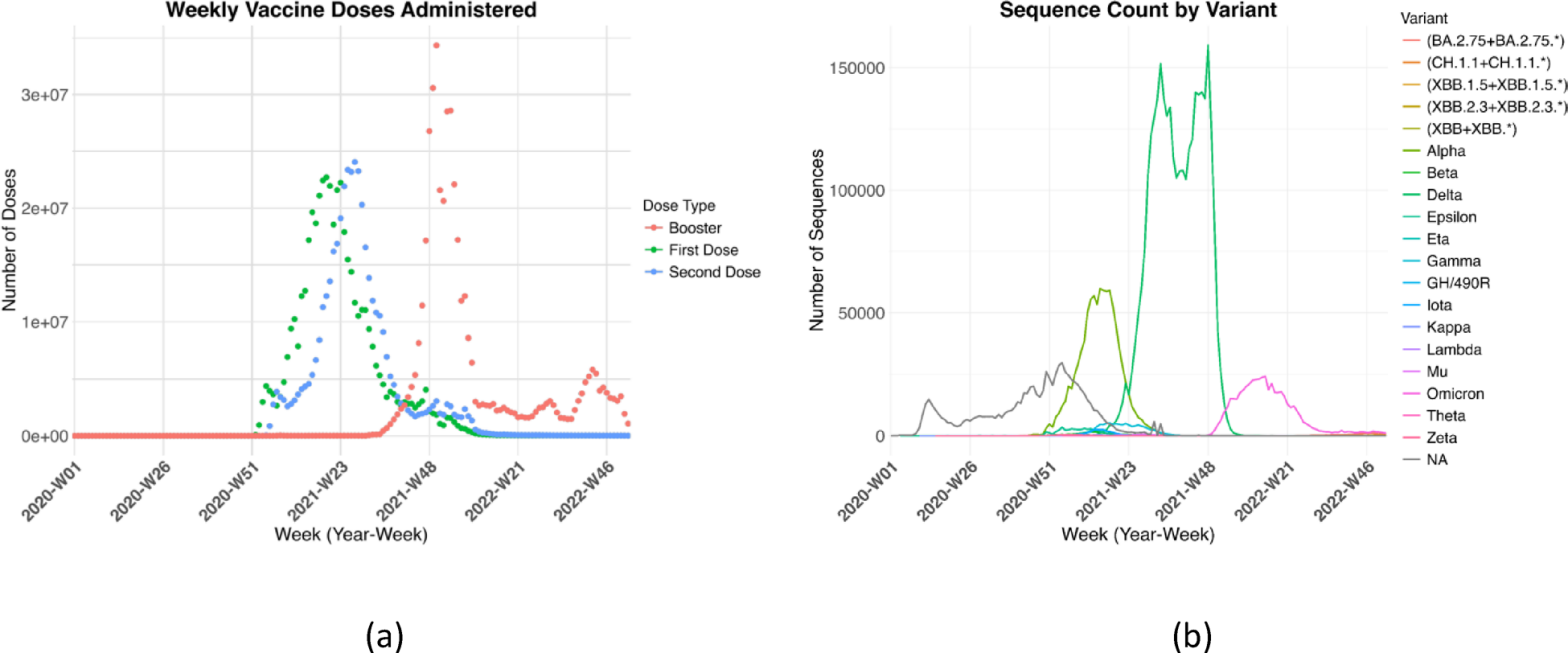
(**a**) Weekly distribution of COVID-19 vaccine doses administered over time, categorized in first dose, second dose, and booster doses (third and fourth doses). Each colored dot corresponds to a different dose type, highlighting the trends in vaccine uptake and the progression of the vaccination campaign over time from 2020 to 2023. (**b**) Number of sequences uploaded to the GISAID database for each COVID-19 variant from 2020 to 2022. Each colored line represents a different variant, showing the number of protein sequences submitted over time.

Our second key data source for our analysis is the European Centre for Disease Prevention and Control (ECDC) repository^14^. The ECDC has been monitoring the progression of COVID-19 across Europe since the initial outbreaks in 2020. It provides weekly reports that include various metrics such as the number of cases, case-to-test ratios, cumulative cases, incidence rates, hospitalizations, mortality rates, and vaccination data, both for the entire European region and for individual countries. While the SI is recorded daily, ECDC data are available on a weekly basis. As a result, for our integrated dataset, we chose calendar weeks as the unit of analysis, aggregating the daily values of the Stringency Indices into 53 weeks for 2020, 52 weeks for 2021, and 52 for 2022. Using weekly data helps mitigate irregularities present in daily records, which are prone to errors and fluctuations due to factors such as inconsistent testing rates and methodologies. However, most of the 29 countries analyzed lacked complete data on hospital admissions and ICU bed occupancy. The selection of the nine nations included in this research was based on prior analyses, which showed that these countries had minimal missing data for the ECDC epidemiological and genetic variables relevant to our study. For our analysis, out of the ECDC datasets’ wide range of features, we selected the following key variables: New weekly case incidence (New Cases), Weekly hospital admissions (admHosp), Weekly ICU admissions (admICU), Weekly death incidence (Deaths), First dose vaccination (First Dose), Second dose vaccination (Second Dose), and three additional doses (DoseAdditional1, DoseAdditional2, DoseAdditional3). The additional doses were aggregated into a single variable, named Booster (Fig. 2A). Each variable was then rescaled by dividing it by the population of the respective country and normalized per 100,000 individuals to allow for meaningful comparisons. For missing data, the imputation method involved replacing missing values (NA) with the last valid entry from the same column, as long as the previous value was neither missing nor zero. If no valid previous value existed, or if the last value was zero, the missing value was replaced with zero. While zero imputation could potentially introduce bias in more sparse datasets, in this case the risk is mitigated because the missing data points in the Nations chosen are a few (median missing value across the different countries is 5.7%) and occur mostly in the early weeks of 2020—when either no records were taken or SARS-CoV-2 was not yet spreading in these countries. Our imputation approach ensures the dataset is complete, reducing the potential bias from incomplete data. At the end of ECDC Preprocessing, we produced nine datasets for each year, each corresponding to a specific country of interest.

#### GISAID

In addition to the epidemiological surveillance variables, we included also the role that variants such as Alpha, Delta, and Omicron played in the pandemic and their impact on the SI. To this end, we conducted an analysis of the frequency of Spike protein sequences in our third key dataset: GISAID, a global initiative that provides open access to genomic data of the influenza and Sars-CoV-2 Viruses. The Spike protein is a key component of a virus, responsible for allowing it to enter human cells^3^. Rapid mutations in the spike-protein gene drive amino-acid changes in the protein itself, generating variants with distinct infectivity and pathogenicity profiles^15^. The original Wuhan strain (NA) was predominant in early 2020. In 2020, the Beta variant (B.1.351, South Africa)^14^, Alpha (B.1.1.7, United Kingdom), Delta (B.1.617.2, India), and Gamma (P.1, Brazil)^16,17^ emerged. In 2021, Omicron (B.1.1.529, South Africa) was identified and became the dominant strain worldwide. Other variants (e.g., Epsilon, Zeta, Kappa) had limited impact (Table 1). Currently, Omicron and its sublineages—such as BA.2.75, XBB, XBB.1.5, and CH.1.1^18,19^—remain the most widespread variants globally (Fig. 2B).

**Table 1:** SARS-CoV-2 variants included in the dataset for each simulation year.

Year	Variants included
2020	Alpha (Al), Beta (Be), Delta (De), Zeta (Ze)
2021	Alpha (Al), Beta (Be), Delta (De), Zeta (Ze), Theta (Th), Gamma (Ga), Epsilon (Ep), Eta (Et), Kappa (Ka), Iota (Io), Mu (Mu), Lambda (La), GH490R (GH), Omicron (Om)
2022	Delta (De), GH490R (GH), Omicron (Om), Alpha (Al), BA.2.75, CH.1.1, XBB, XBB.1.5

The table presents the variants considered in the models for 2020, 2021, and 2022, highlighting the increase in variant diversity in 2021 and the subsequent reduction in 2022, with the emergence of new subvariants such as BA.2.75, CH.1.1, XBB, and XBB.1.5

For our analysis, we downloaded 16,187,950 Spike protein sequences from GISAID, spanning from December 24, 2019, to November 8, 2023, along with their corresponding lineages. To enhance data quality, we implemented several filtering criteria. Firstly, we retained sequences only from specific countries and excluded any sequence that contained missing or unrecognized amino acids. Additionally, we selected sequences whose lengths were within ± 30 amino acids of the median length for their lineage, aligning with the expected Spike protein length of 1241 to 1301 amino acids, resulting in a dataset with 4,506,983 Spike proteins. Finally, we focused on sequences and metadata whose collection dates were included in the period of our analysis. Subsequent filtering was conducted to focus on sequences from the nations of interest in this research. For each pair of countries and variants we calculated the variant frequency as a fraction of the total sequences uploaded to GISAID for the variant on a weekly basis (using calendar weeks) during the specified year. This process resulted in the creation of nine distinct datasets—one for each country for each year of the simulation—each containing the weekly percentage of observed variants. The variants, during our analysis period, did not appear uniformly across all countries^20^, due to genetic differences or limited sequencing capacity. To maintain data consistency, we imputed any missing variant information for each year with zeros, ensuring the same set of features within our model and facilitating cross-country comparisons. This approach implicitly assumes that if a variant was not sequenced in a given country, it did not meaningfully affect that country. While this assumption has inherent limitations, it is partially supported by the fact that European countries account for approximately 48.05% of the sequences uploaded to GISAID, suggesting robust sequencing coverage in this region.

### Multilevel autoregression model

#### Correlation analysis

To assess potential relationships between variables, the correlation between the SI and each epidemiological and genetic variable has been assessed for each year analyzed (i.e from 2020 to 2022) using Pearson’s linear correlation coefficient, that can be written as follows:

1 $$\rho = \frac{{Cov\left( {X,Y} \right)}}{{\sigma_{X} \sigma_{Y} }}$$

where X and Y are two numerical variables, $Cov\left( {X,Y} \right)$ indicates their covariance and $\sigma$ indicates the variance. The score can range from − 1 to 1. A positive value indicates an increase in one variable is associated with an increase in the other variable, while a negative score indicates that the two variables grow in opposite directions. 0 indicates an absence of correlation.

The results are presented in Table 2.

**Table 2:** The contemporaneous bivariate correlations between each index and the weekly epidemiological and genetic variables for 2020 were analyzed across all countries.

	New cases	admHosp	admICU	Deaths	First dose	Second dose	Booster	Alpha	Beta	Delta	Zeta
SI	**0.33**	**0.41**	**0.42**	**0.48**	**0.11**	7e − 5	0.08	**0.27**	0.07	0.08	0.03

Significant results (*p* < 0.05) are highlighted in bold. Correlations were calculated by combining data from all available countries into a single vector for each variable.

Table 2 shows that in 2020 SI was significantly and positively correlated with all epidemiological variables, especially with admissions to hospitals and ICU, and deaths, which have the highest correlations scores. Concerning the other variables, among all variants, only the Alpha has a significant, although weak, correlation, while the others, which appeared at the end of the year, appear not to be correlated to policy intensity. First vaccine doses, which began towards the end of the year, also have a significant correlation, although the low score indicates a substantial absence of association. The correlations between each index and the weekly epidemiological and genetic variables for 2021 and 2022 are reported in Tables S1 and S2, respectively.

#### Population model design

From our correlation analysis, we identified the relationships between the Stringency Index (SI) and various epidemiological and genetic variables. To further investigate these relationships, we implemented a multi-country autoregressive predictive model for each simulation year^9,21^ incorporating both policy and epidemiological data. Autoregressive models inherently incorporate time lags to capture temporal dependencies within the data. This allows us to assess how past values influence current trends. To determine the optimal magnitude of the time lag, we tested various time lags and selected the best one based on the Akaike Information Criterion (AIC), whose formula is provided in Eq. (2):

2 $${\text{AIC}} = {2}k - {\text{2 ln}}\left( L \right)$$

where k represents the number of parameters estimated by the model, and L is the maximum likelihood of the model.

In detail, for each simulation year, we applied Multilevel Vector Autoregression (mlVAR) to construct an integrated model that could be used across all included countries. This method allows for the analysis of temporal dependencies while accounting for variations between different entities—in this case, European countries. Multilevel Vector Autoregression (mlVAR) is a statistical modeling technique specifically designed to analyze a population of related entities while accounting for both shared characteristics and individual variations^22,23^. Rather than assuming a single, uniform model that applies identically to all countries, mlVAR adopts a hierarchical structure that allows for country-specific differences while still maintaining a central population-wide framework. In this approach, each country is treated as an individual unit within a broader population, with its own set of model parameters. These country-specific models are not completely independent but are instead slight variations of a central population model. The central model captures the overall trends and patterns that are common across all countries, while the individual country models introduce deviations that account for unique national characteristics, such as differences in policy responses, healthcare infrastructure, and public compliance with restrictions. This hierarchical structure enables mlVAR to balance generalizability and specificity,ensuring that the model captures overarching relationships among epidemiological, policy, and genetic variables across multiple countries, while also allowing for country-specific nuances that influence the trajectory of the pandemic in each region. By doing so, mlVAR provides a more accurate and flexible representation of time-dependent processes across different geographical contexts.

To implement this approach, we modeled each of the nine selected European countries as individual entities within a broader population framework. The population-wide effects are captured as Fixed Effects (FE), which include parameters that remain consistent across all countries. Equation (3) illustrates the fixed effects calculation, setting the Stringency Index as the dependent variable as an example.

3 $$\begin{aligned} {\mathbf{I}}\left( t \right) = & \beta_{01} + \beta_{11} \cdot I(t - 1) + \beta_{21} \cdot NewCases(t - 1) + \beta_{31} \cdot admHosp(t - 1) \\ & + \beta_{41} \cdot admICU(t - 1) + \beta_{51} \cdot death(t - 1) + \beta_{61} \cdot FirstDose(t - 1) \\ & + \beta_{71} \cdot SecondDose(t - 1) + \beta_{81} \cdot BoosterDose(t - 1) + \beta_{91} \cdot Alpha(t - 1) \\ & + \beta_{101} \cdot Beta(t - 1) + \beta_{111} \cdot Delta(t - 1) + \beta_{121} \cdot Zeta(t - 1), \\ \end{aligned}$$

On the other hand, Random Effects (RE) account for country-specific deviations from the central model. These deviations are assumed to follow a Gaussian distribution, where the mean represents the estimated population average, and the standard deviation reflects the extent of country-specific variations (Eq. 4).

4 $$\beta_{ij} \sim N\left( {\beta_{j,pop} ,\,\sigma_{\beta j,pop} } \right)$$

Additionally, Eq. (3) conforms to the definition of a multivariate autoregressive (AR) model, in which the variables at time t depend solely on their values at the previous time step (t − 1). In statistical terms, an AR model represents a random process where the output variable is a linear function of its past values, with an additional stochastic term to account for uncertainty. This results in a stochastic difference equation, commonly used to model time-dependent phenomena.We implemented the multilevel vector autoregression model using RStudio, leveraging the dedicated R packages mlVAR^24^ and lme4^25^. The input data used for estimating model parameters consists of:

- Stringency Index (SI)
- Four epidemiological parameters: new cases (nCnC), hospital admissions (aHaH), ICU admissions (aICUaICU), and deaths (dd)
- Three vaccination-related variables: first dose (fDfD), second dose (sDsD), and booster dose (bDbD)
- Variant frequency columns, which detail the presence of specific SARS-CoV-2 variants for each simulation year (Table 1)
- Country ID, identifying the country associated with each dataset

Each simulation year generates nine datasets—one for each country—ensuring that country-specific patterns and trends are accurately represented in the model.

The primary output of the model consists of the estimated regression parameters for the autoregressive model. Equation (5) provides an example of this estimation for SI (indicated just with “I” for simplicity) in 2020, where the conditional component accounts for the Random Effects, meaning the contribution depends on the country ID. A more detailed model for the 2020 analysis is provided in Eq. (1) of the supplementary file. By structuring the model in this way, we capture both the overarching trends common to all countries and the country-specific deviations that influence epidemiological and policy-related dynamics. This hierarchical structure makes mlVAR a powerful tool for studying cross-country variations in pandemic responses and outcomes while preserving the temporal dependencies essential to autoregressive modeling.

5 $$\begin{gathered} I\left( t \right) \sim I(t - {1}) + NewCases(t - {1}) + admHosp(t - {1}) + admICU(t - {1}) \hfill \\ \quad + \,death(t - {1}) + newCases\left( t \right) + admHosp\left( t \right) + admICU\left( t \right) + death\left( t \right) \hfill \\ \quad + \,FirstDose(t - {1}) + SecondDose(t - {1}) + BoosterDose(t - {1}) + Alpha(t - {1}) \hfill \\ \quad + \,Beta(t - {1}) + Delta(t - {1}) + Zeta(t - {1}) + NewCases\left( t \right) + admHosp\left( t \right) \hfill \\ \quad + \,admICU\left( t \right) + death\left( t \right) + FirstDose\left( t \right) + SecondDose\left( t \right) + BoosterDose\left( t \right) \hfill \\ \quad + \,Alpha\left( t \right) + Beta\left( t \right) + Delta\left( t \right) + Zeta\left( t \right) + (({1}|ID) + (I(t - {1})|ID) \hfill \\ \quad + \,(NewCases(t - {1})|ID) + (admHosp(t - {1})|ID) + (admICU(t - {1})|ID) \hfill \\ \quad + \,(death(t - {1})|ID) + (FirstDose(t - {1})|ID) + (SecondDose(t - {1})|ID) \hfill \\ \quad + \,(BoosterDose(t - {1})|ID) + (Alpha(t - {1})|ID) + (Beta(t - {1})|ID) \hfill \\ \quad + \,(Delta(t - {1})|ID) + (Zeta(t - {1})|ID)) \hfill \\ \end{gathered}$$

## Results

### Magnitude time lag selection

**Fig. 3:**
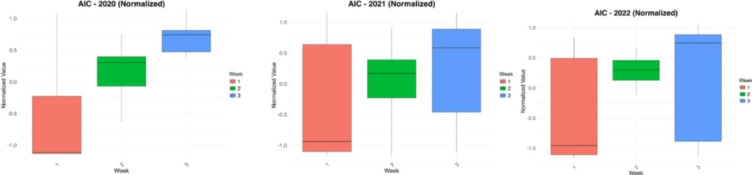
Normalized AIC for the model applied to all three years with a lag of one, two and three weeks. It can be seen that the model performs better with a lag of one week in 2020 (Kruskall–Wallis *P*-value = 0.008). No significant differences are visible for 2021 and 2022, although the AIC is generally still lower with a one-week delay.

After a dedicated analysis and thorough considerations, we selected a one-week lag for the mlVAR model. This choice was motivated by both theoretical considerations about COVID-19’s epidemiological dynamics and empirical evidence from model selection criteria. First, while the incubation period of SARS-CoV-2 can range from 2 to 14 days^26,27^, policy effects on key epidemiological indicators (e.g., case rates, hospitalization rates) typically begin to manifest within approximately one week. In fact, several empirical studies demonstrate that non-pharmaceutical interventions can yield measurable shifts in key epidemiological indicators within roughly one week. Reported case-growth fell within 1–5 days of stringent social-distancing orders in U.S. counties^28^ statewide mask mandates lowered daily case growth during the first 1–20 days after implementation^29^; large-scale anti-contagion policies produced a significant reduction in infection-growth during the “first week” post-enactment across six countries^30^; and early community-mitigation packages generated discernible inflections in case trends only a few days after adoption^31^. Even hospitalization growth rates declined by ~ 3 percentage points during the first two weeks following statewide mask mandates^32^. This timescale reflects not only the incubation period but also the reporting and testing behaviors, which often align with weekly cycles in data collection. Thus, a one-week lag strikes a balance between capturing the immediate, short-term response to containment measures and avoiding overfitting that can arise from setting longer lags. Second, we compared one-, two-, and three-week lags empirically by computing the AIC for each model (Fig. 3). Although the improvement in AIC from one- to two-week lags was statistically significant only in 2020, the one-week lag consistently yielded the best or near-best performance across the three years, indicating that it is an adequate timescale for detecting policy effects on the analyzed epidemiological variables. Maintaining a uniform one-week lag across all years also provides continuity within the different analyzed years and with our previous work^9^, facilitating comparisons over time. Taken together, these theoretical and empirical considerations support our decision to use a one-week lag in the mlVAR analysis.

### Fixed effects

Fixed effects in the mlVAR model represent the relations that each variable has with all the other variables considering the entire population, i.e. all countries. We performed the analysis for each year of the pandemic, obtaining different fixed effects, as the conditions changed in time with the introduction of vaccinations and new variants. Detailed results for each year are reported in the following sections.

#### 2020

**Fig. 4:**
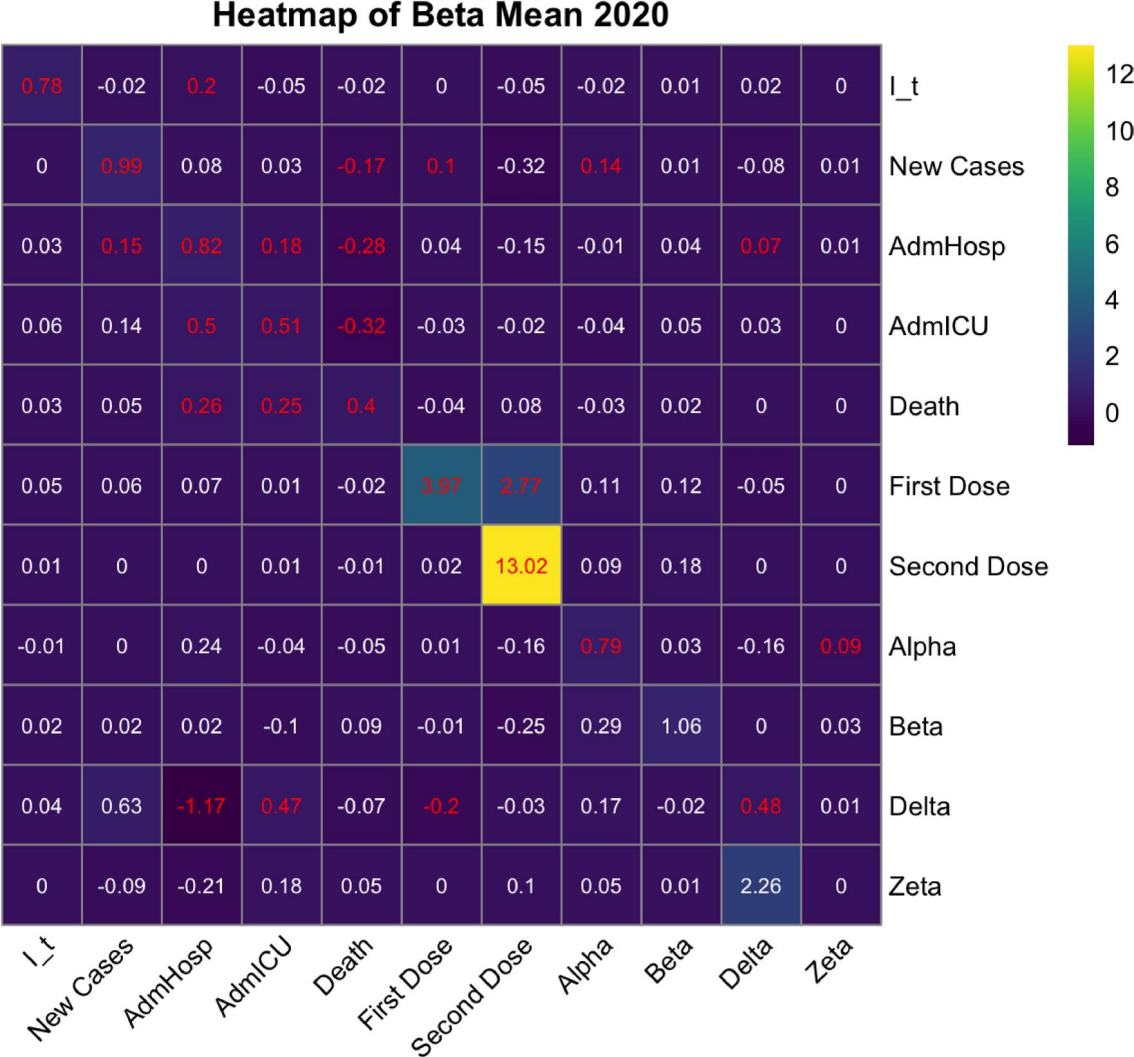
Heatmap of the fixed effects’ coefficients for the year 2020. The significant ones (*P*-value < 0.05) are highlighted in red. The rows indicate the variables at time *t*, i.e. the outcomes, whereas the columns indicate the variables at time *t* − 1, i.e. the covariates. For instance, in the first row, I_t is the outcome variable, while each column (I_t, New Cases, AdmHosp, AdmICU, Death, First Dose, Second Dose, Alpha, Beta, Delta, Zeta) represents a covariate used by mlVAR to predict this outcome.

When the virus spread in Europe for the first time in 2020, vaccinations were not available, and the original strain and the Alpha variant were causing most of the infections. Towards the end of the year, new variants arrived and the first vaccinations were made. Figure 4 shows a heatmap of the Beta coefficients for all the relations between all combinations of variables and a time lag of one week where the rows correspond to the outcome variables at time t, while the columns represent the covariates at time t − 1, which mlVAR uses to predict the outcomes.

From this figure, looking at the significant relations, it can be noticed that the Stringency index in each week of 2020 strongly depends on its own value in the previous week, and is also positively influenced by the rate of hospitalizations. The epidemiological indicators (cases, hospitalizations and deaths) also have some influence on each other in time, for example an increase in new cases is associated with an increase in hospitalizations, ICU occupancy and deaths after one week, and an increase in all hospitalizations is associated with a subsequent increase in ICU occupancy. In the same way, deaths tend to increase after an increase in hospitalizations, but the increase in deaths is associated with a decrease in hospital occupancy after one week. Vaccination data also shows some significant coefficients, however their relevance is probably low as vaccinations started at the end of the year, therefore the data points are not sufficient to show a significant trend.

#### 2021

**Fig. 5:**
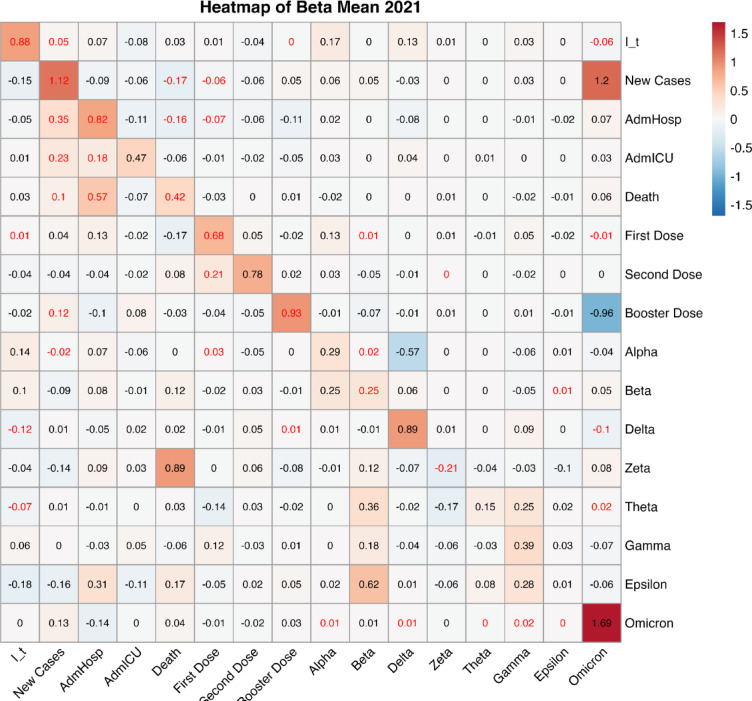
Heatmap of the relevant fixed effects’ coefficients for the year 2021. The significant ones (*P*-value < 0.05) are highlighted in red. The rows indicate the variables at time *t*, i.e. the outcomes, whereas the columns indicate the variables at time *t* − 1, i.e. the covariates. For instance, in the second row, New Cases is the outcome variable, while each column (I_t, New Cases, AdmHosp, AdmICU, Death, First Dose, Second Dose, Alpha, Beta, Delta, Zeta, Theta, Gamma, Epsilon,Omicron) represents a covariate used by mlVAR to predict this outcome.

The central year of the pandemic was the most eventful one, as several waves of infections and new variants came in a period in which vaccinations were becoming gradually more diffuse. Figure 5 displays the heatmap of the most relevant mlVAR fixed effects for the year 2021, including variants that played a significant role during that period. Besides these variants, the analysis includes the stringency index, vaccination data, including the booster dose, and key epidemiological variables. For a complete view of all fixed effects, refer to Fig. S1. As in previous figures, the rows represent the outcome variables at time t, while the columns denote the covariates at time t − 1, which mlVAR utilizes to predict the outcomes. Looking at the significant coefficients, highlighted in red, several interesting relations can be noticed. First of all, the stringency index appears to increase following an increase in new cases and hospitalizations (which, considering the magnitude of the coefficient, still appear to be the strongest epidemiological factor), excluding the ICU ones. Vaccinations have an impact on all epidemiological variables, as all hospitalizations and new cases decrease with an increase of some vaccination dose, in particular first doses are associated with a decrease of new cases and hospital admissions, booster doses are associated with a decrease in hospital and ICU admissions, second doses are associated with a decrease in all of the three variables.

Looking at the coefficients associated with the SI, the one related to ICU admissions appears in contrast with the others, since it seems to indicate that an increase in ICU hospitalizations can cause a reduction of policy stringency. However, it has to be considered that this result could be influenced by the natural lag between the infection and the worsening of the disease, therefore it is likely that new cases and general hospital admissions were considered as more meaningful indicators of the increasing spreading of the virus.

Some interesting relationships can be noticed also concerning the variants: the Alpha variant, prevalent between the end of 2020 and the beginning of 2021, is negatively influenced by the increase of the Delta variant, that rapidly became prevalent in 2021, replacing Alpha in a relatively short time. Plus, the Omicron variant seems to be related to a notable increase in new cases, with a positive, significant and relatively high coefficient.

#### 2022

**Fig. 6:**
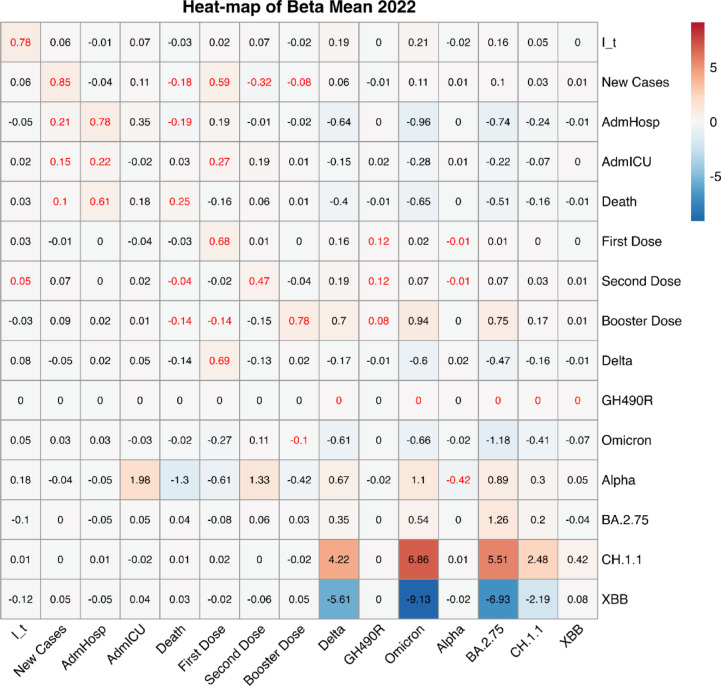
Heatmap of the fixed effects’ coefficients for the year 2022. The significant ones (*P*-value < 0.05) are highlighted in red. The rows indicate the variables at time *t*, i.e. the outcomes, whereas the columns indicate the variables at time *t* − 1, i.e. the covariates. As an example, in the Third row, I_t is the outcome variable, while each column (I_t, New Cases, AdmHosp, AdmICU, Death, First Dose, Second Dose, Booster Dose, Delta, GH490R, Omicron, Alpha, BA.2.75, CH.1.1, XBB) represents a covariate used by mlVAR to predict this outcome.

Figure 6 shows the fixed effects of mlVAR applied to the year 2022, i.e. the final full year of pandemic. Consistently with the previous figures, the rows correspond to the outcome variables at time t, while the columns represent the covariates at time t − 1, which mlVAR uses to predict these outcomes. In this time frame, restrictions around the world were gradually lifted. Thanks to the increasing immunity of the population due to the combined effects of vaccinations and previous exposures, and to the diffusion of the Omicron variant that seemed to be less severe although highly contagious^33^, hospitalizations and deaths showed a decreasing trend. As a consequence, the stringency index in 2022 appears not to be related with any epidemiological variable. Some temporal dependencies are still present for the epidemiological variables, and some significant coefficients can be noticed also concerning the variants, however considering that the variant variables depend on the percentage of genetic sequences identified, we do not believe that they represent meaningful results.

### Random effects

**Fig. 7:**
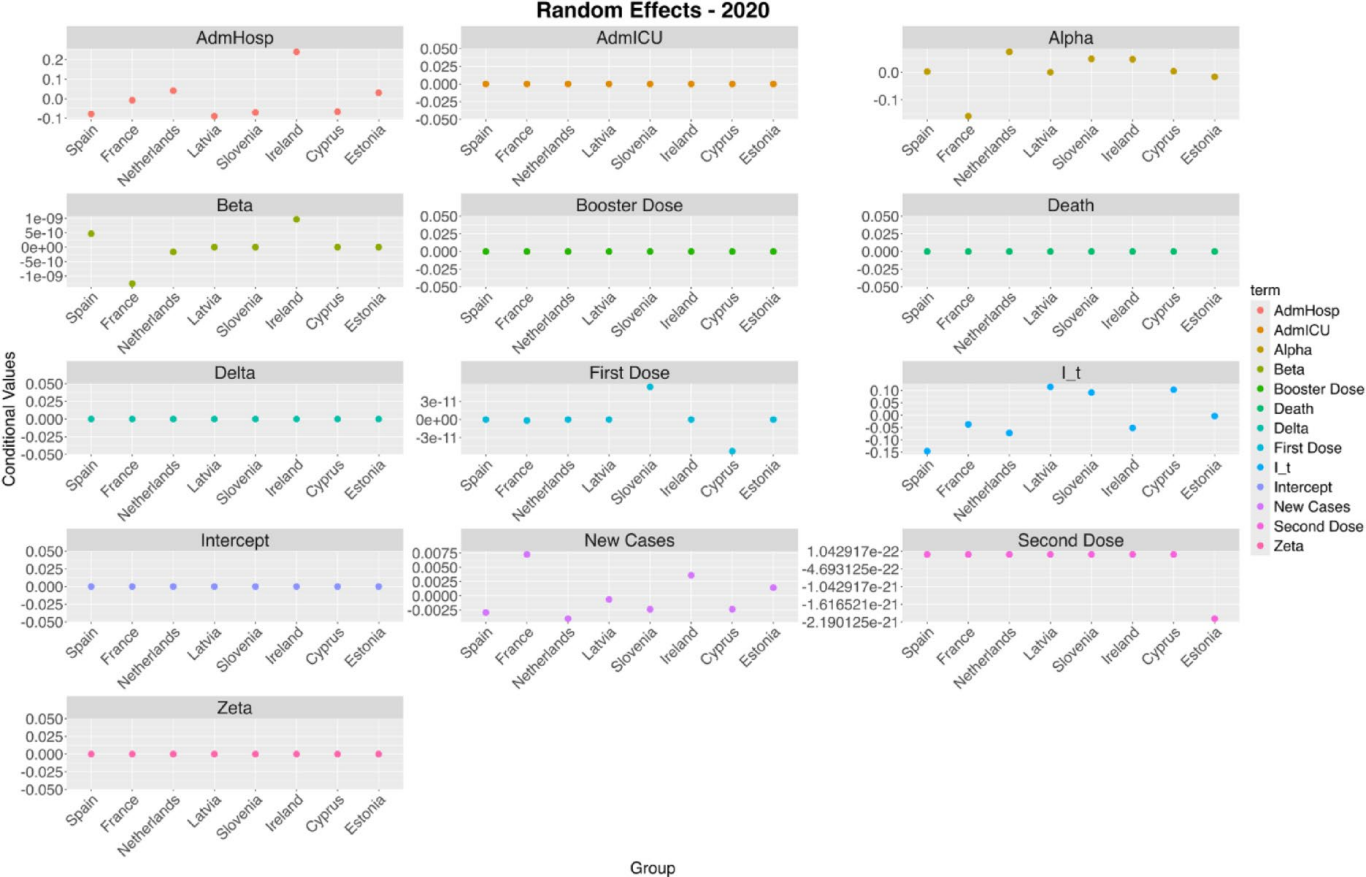
Random effects (country-wise variation on the model intercept) for all predictors with stringency index as outcome in 2020.

While fixed effects describe the general relations between variables in the entire population, random effects describe the individual variations of the model outcome for each of the countries and allow to take into account the variability due to each country’s own socio-demographic features. Figure 7 shows the random effects of the 2020 model for each variable, considering the stringency index as the dependent variable. Random effects of the 2021 and 2022 models are reported in Figs. S2 and S3 in the online appendix section. Some interesting phenomena can be seen analyzing these figures together with the fixed effects results. For example, according to the fixed effects in 2020 the stringency index was significantly influenced only by its previous value and the increase of admissions to hospitals, however the random effects show that this influence was higher in some countries such as Ireland, but slightly lower in Spain, Slovenia, Latvia and Cyprus.

In 2021, on the contrary, the effect of hospitalizations on the index is constant for all countries, whereas some other variables show higher variation, for example new cases seem to have a greater impact on the stringency index in the Netherlands than in Spain or Ireland. Some country-specific differences are present also in 2022, however the only significant fixed effect is the one related to the previous stringency index, which appears to be the highest in Latvia, Slovenia and Cyprus and the lowest in Spain.

### Temporal progression of the coefficients

**Fig. 8:**
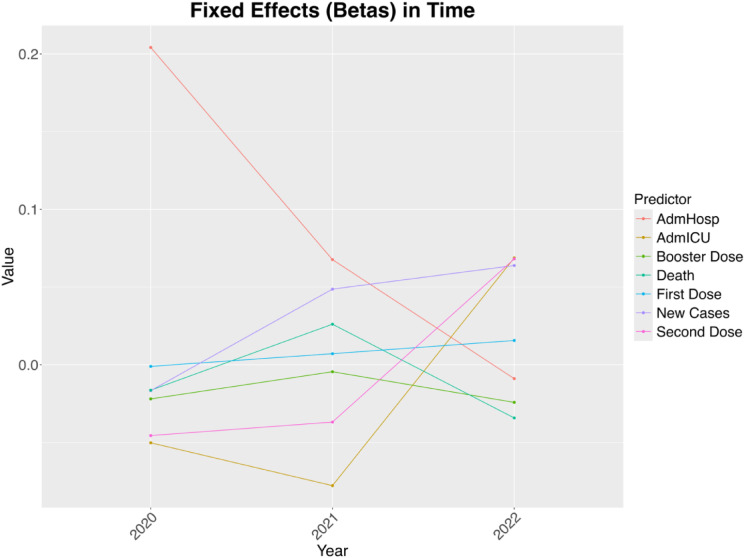
Variations of the fixed effects coefficients indicating the effects of the epidemiological variables on the stringency index in the three considered years.

A useful representation of how the general mutual impacts between the epidemiological variables and the policies varied in all the considered countries can be obtained plotting the value progressions of the fixed effects coefficients in the three years of pandemic. Figure 8 shows this variation setting the stringency index as the dependent variable. Observing this figure, it can be noticed that hospital admissions is the variable with the highest influence on the stringency index in the first two years of pandemic, especially in the first phase in 2020, when the deadliest variants were causing most of the infections and vaccines were not yet available. In 2021, with the increasing vaccinated population and new variants characterized by high infection power but relatively lower hospitalization rates, hospitalization rates tend to have less influence on the creation of stringent policies, whereas new cases gain importance. Another interesting observation can be made looking at the Second Dose coefficient, that is negative in 2020 and 2021, meaning that in general the increase in second dose administrations is followed by a relaxation of the policies. The coefficient increases in 2022, however from the heatmap it can be seen that this number is not significant, as the number of people who had received the second dose in 2022 is high and constant.

Considering the stringency index as covariate instead of outcome, interesting inter-dependencies can be seen as well. Figure S4 shows the betas variation in time setting the number of new cases as the dependent variable. Interestingly, an increase in the stringency index does not appear to reduce new cases in 2020, as shown also in the heatmaps, but it seems to become more effective in 2021, whereas the 2022 value appears to be not significant. Interesting insights can be noticed looking at the vaccination data: vaccines appear to significantly reduce new cases only when the percentage of vaccinated population is relatively high, in fact it can be seen that the coefficients are negative and significant in 2021 for first and second dose, and in 2022 for the booster dose. The booster dose, however, significantly reduced hospitalizations also in 2021, as shown in Fig. 5. It should be noted that the relatively high coefficient for the ”first dose” and “second dose” variables in 2022 is not reliable, as in 2022 the rate of population who had received those doses was high and constant.

## Discussion

Being caused by a highly contagious and previously unknown virus, the COVID-19 pandemic quickly became a world crisis that lasted for three years. Finding themselves suddenly burdened by a high load of hospitalizations, governments and local political entities decided to establish containment policies aiming at reducing social gatherings, national and international travel, and business activities that could be the ground for new outbreaks. Since the pathogen was unknown, at the beginning of the pandemic most of these policies were designed as a reaction to the public health situation rather than as the result of a data-driven analysis, and had numerous side effects such as socioeconomic and mental health impacts. Identifying the best policies can help create unified protocols, aiming at facing future pandemic threats via optimized interventions, and maximize effectiveness while limiting the negative social and economic impacts. Previous studies^10^, including ours^9^, focus only on the initial phase of the pandemic, and they do not take into account the presence of different virus variants, nor how the vaccinations influenced the choice of the policies. Other studies on these topics focus on limited geographical areas. In general, past research showed that the policy effectiveness has been rather heterogeneous depending on the geographic regions considered, although, not surprisingly, the most effective ones on average seem to be those related to increasing health monitoring and resources. In this work, we aimed at providing a framework to analyze the entire course of the pandemic, with a series of models showing the delicate dependencies among the epidemiological and sociopolitical aspects of the pandemic, considering that those two aspects are enclosed in a mutual exchange of causal factors and are characterized by a spatial dimension that works on different levels. To the best of our knowledge, this is the first work to provide a complete analysis that includes both the population-level effects and the individual ones for the entire duration of the pandemic, which includes several transition phases where health policies were combined with vaccination strategies to contrast the virus, and at the same time were countered by the advent of new variants.

The general results of our analysis show notable differences in the variable relations in time, but a certain agreement among the different countries considered. In particular, it seems that in 2020, when the virus was new and most lethal, the hospitalizations rate was the indicator more associated with stringent policies, which were probably a reaction to the increasing pressure on the health systems. This happened more in Ireland than in other countries, whereas the impact was relatively lower in Slovenia, Latvia, Spain and Cyprus. Increasing hospitalization rates were followed by more and more stringent countermeasures. However, the stringency increase did not lead to a significant decrease in hospitalizations after one week, but it should be noted that this relation would probably be different considering a larger time frame. From 2021, “new cases” became another epidemiological variable associated with higher policy stringency. This probably happened mainly for two reasons: on one hand, new variants with a higher infective power became prevalent, causing new cases to rise dramatically. Not knowing these new pathogens and fearing a new increase in hospitalizations, policy makers acted based on this case increase. On another hand, tests for COVID were much more common than at the beginning of the pandemic, allowing the identification of new cases much faster and in a greater number. In this situation, the coefficient is the lowest for Ireland and Spain, whereas the relation is higher in the other countries, especially the Netherlands. Differently from 2020, the reverse relation is significant, i.e. an increase in stringent policies is associated with a significant decrease in new cases. This is not true for 2020, although we would argue that new cases were not properly tracked, and this might have strongly contributed to the lack of statistical significance. Vaccinations are generally significantly associated with a reduction of cases and hospitalizations, but with different dynamics depending on the coverage of each dose. In fact, the first dose is most effective in 2021, whereas second and booster doses, which began to be administered in 2021, are more effective in 2022, suggesting that the overall effect on the country’s new case and hospitalization rates becomes visible when a certain percentage of the population is vaccinated, although the precise percentage is difficult to identify with our data. However, only an increase of second doses in 2021 is associated with a decrease in policy stringency in all countries. Concerning the sequenced variants, the only variant that is significantly associated with policy making was Delta in 2021, as a higher number of sequences is followed by higher stringency. The rising of the Omicron variant is related to an impressive spike in new cases in 2022, but an increase in hospitalizations or policy stringency does not seem to follow in any of the considered countries.

Arguably, this study presents several limitations, mainly consequential of the implicit limitations of the epidemiological data, i.e., the collected data does not necessarily represent the real situation. Collected data depend on the quantity and frequency of tests and their collection methods, as well as variant sequencing that were performed by a specific country. Even though the hierarchical nature of the model is a point of strength, as it considers both the entire set of countries and the individual ones, the geographical model granularity is still very low, as local regions and provinces could have separate epidemiological and sociopolitical situations. This could lead also to incidental correlations, some of which are clearly observed in this study (e.g. first doses and new cases in 2022). It should be also remembered that statistical correlations, although indicative of significant associations between variables, do not prove the presence of causation, therefore this model does not provide direct information about causative effects. Despite these limitations, this study shows a thorough analysis of the interplay between virus spread, restriction policies, vaccination strategies, and viral variants. Most of the epidemiological insights provided by the results were already known (e.g. a higher policy stringency associated to increasing hospitalizations in 2020), however this model has shown the capacity to discover some less known associations, such as the one between second doses and policy stringency reduction, and to quantify heterogeneous relations providing a multi-level, complete analysis including both the population-level (all countries) effects and the individual-level ones (a single country). This tool is easily scalable to other geographical entities and can be potentially applied to new settings, in view of optimizing the policy making process in preparation for new epidemics. Looking at the findings from a practical and interventional point of view, with our past study we demonstrated the importance of enhancing health monitoring and deployment of health resources to contain the effects of the pandemic, these include rapid testing and thorough data collection. In the study reported in this paper, we also showed that the same multilevel approach that we used in the previous study can be expanded to include new data and variables, characterizing the complex links between many aspects of the pandemic, even those that can evolve overtime. In case of future epidemics, this approach could be easily adapted and used to assess the interplay among different factors and their mutual geographic effects, in order to take informed decisions on the possible containment and epidemiological intervention policies. Therefore, a timely intervention by the policy makers aiming at improving health monitoring and data collection, joined with the application of a flexible analysis framework like the one presented here, can help optimize the policy making process, reducing the time needed to gather the necessary information about the disease and finding the epidemiological dynamics that need to be targeted in order to contain the public health effects as fast as possible.

## Conclusion

The COVID-19 pandemic has been the largest public health crisis of the last century so far, which lead governments and public health authorities to establish a variety of containment policies in the attempt of reducing the virus circulation. In a time span of three years, these policies came to a complex interplay with the emergence of new virus variants, a continuous change of the epidemiological landscape, and an increase of vaccination coverage. In this study, we propose a framework to assess such complex interplay, using a vector autoregressive mixed-effect model on data collected throughout the entire pandemic time span in nine European countries: Spain, France, Netherlands, Latvia, Slovenia, Greece, Ireland, Cyprus, Estonia. Results show that the mutual association of the epidemiological variables and the policies’ stringency varied significantly over the course of the pandemic. Policy stringency was strongly associated with the increase in hospitalizations during the first year, whereas the number of new cases became the most associated variable during the following years, with the increase of vaccinations and the advent of new variants characterized by higher infectivity but lower hospitalization rates. All vaccine doses seemed to be effective in reducing new cases and hospitalizations, although the second dose was the only one that significantly associates with a reduction of policy stringency. Finally, new variants had a strong association with several epidemiological aspects, but not with policy stringency. Small differences can be noticed across different countries. Although this approach has some limitations due to the nature of the collected data and the low geographical granularity, this model provides deeper insights on the complex inter-dependencies that were generated across different entities during the COVID-19 pandemic, and represents an easily generalizable framework that can be applied to improve the policy making process in case of future epidemics, considering both the global and the local dimensions.

## Electronic supplementary material

Below is the link to the electronic supplementary material.

Supplementary Material 1

## Data Availability

The datasets generated and/or analysed during the current study and the related codes are available in the COVID19-Europe-Policy-Vaccine-Variants-Analysis repository (https://github.com/simoRancati/COVID19-Europe-Policy-Vaccine-Variants-Analysis/tree/main).
