# Supplementary material for: A comparative analysis of the role of containment policies, vaccination strategies and virus variants in the COVID-19 pandemic across nine European countries
Source: Sci Rep. 2025 Aug 8;15:29109. doi: 10.1038/s41598-025-10132-9 (PMC12334650; doi:10.1038/s41598-025-10132-9)
Supplement: Supplementary file 1 — Supplementary Material 1 [file 41598_2025_10132_MOESM1_ESM.pdf]

A Supplementary

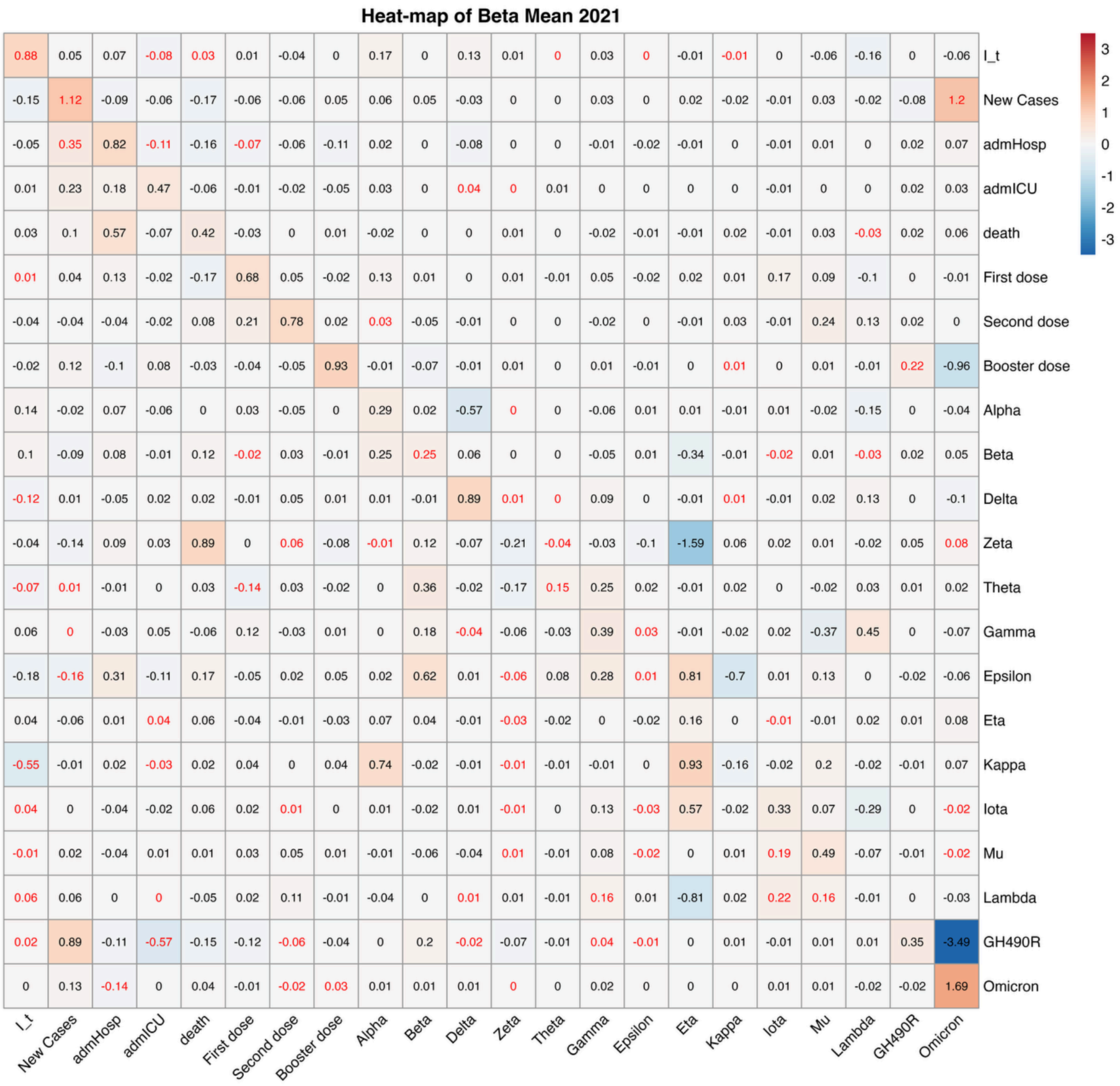

Figure S1: Heatmap of the relevant fixed effects' coefficients for the year 2021. The significant ones (P-Value < 0.05) are highlighted in red. The rows indicate the variables at time t, i.e. the outcomes, whereas the columns indicate the variables at time $t-1$, i.e. the covariates. For instance, in the second row, New Cases is the outcome variable, while each column (I_t, New Cases, AdmHosp, AdmICU, Death, First Dose, Second Dose, Alpha, Beta, Delta, Zeta, Theta, Gamma, Epsilon, Eta, Kappa, Iota, Mu, Lambda, GH490R, Omicron) represents a covariate used by mlVAR to predict this outcome.

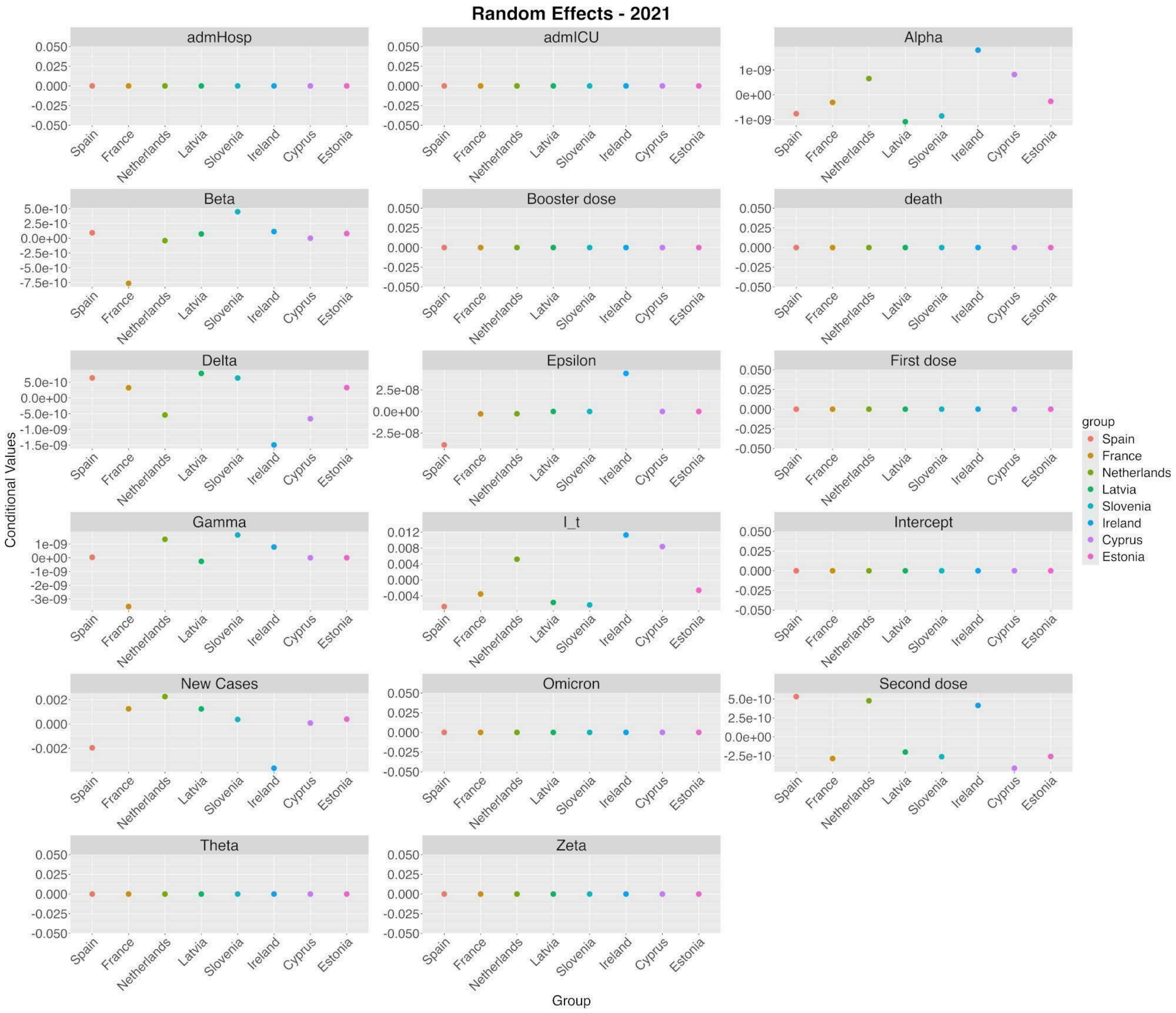

Figure S2: Random effects (country-wise variation on the model intercept) for all predictors with stringency index as outcome in 2021.

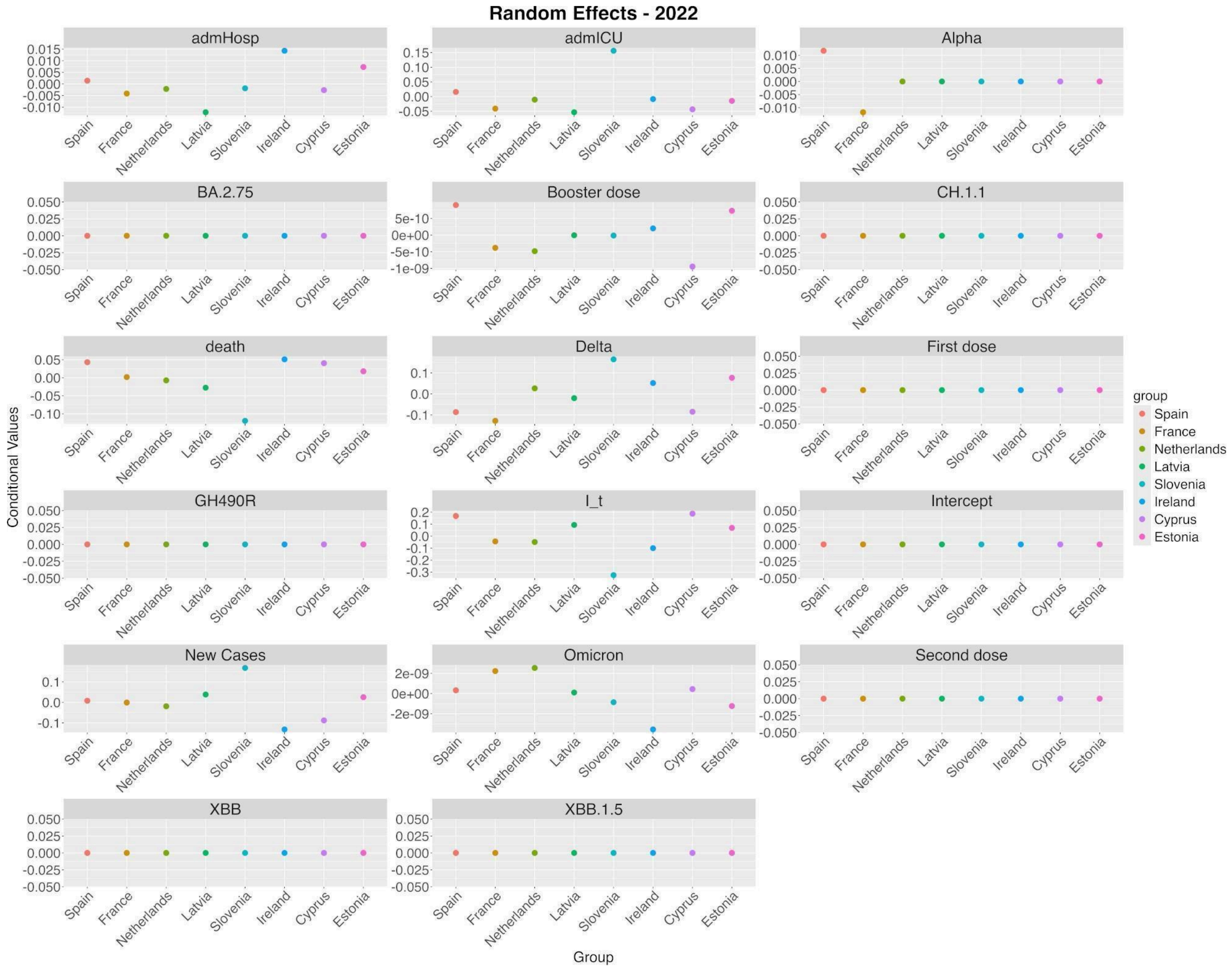

Figure S3: Random effects (country-wise variation on the model intercept) for all predictors with stringency index as outcome in 2022.

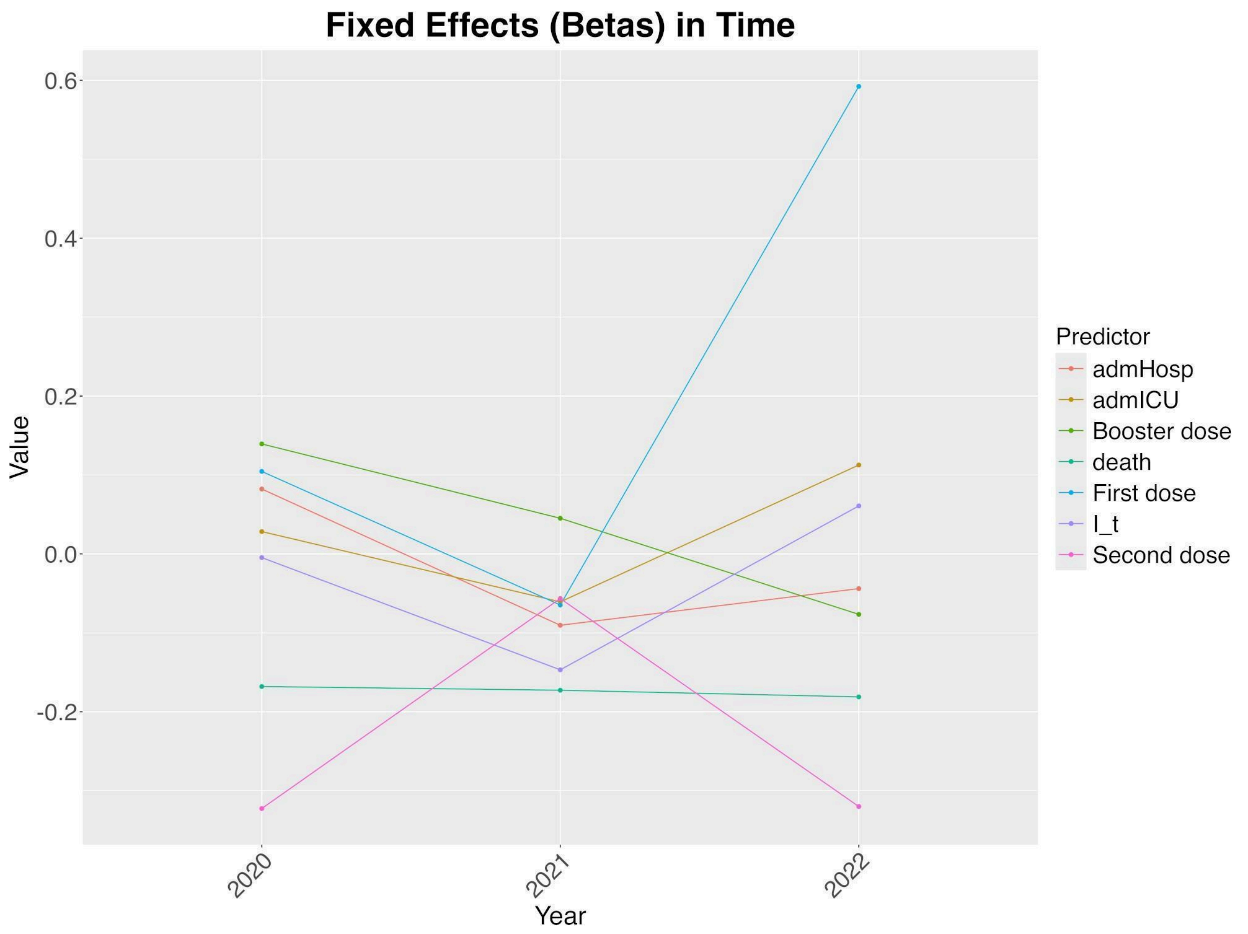

Figure S4: Variations of the fixed effects coefficients indicating the effects on new cases of the other epidemiological variables and the stringency index in the three considered years.

Supplementary Table S 1: The contemporaneous bivariate correlations between each index and the weekly epidemiological variables for 2021 were analyzed across all countries. Significant results ($p < 0.05$) are highlighted in bold. Correlations were calculated by combining data from all available countries into a single vector for each variable.

	SI
New Cases	-0.04
adm_Hosp	**0.17**
adm_ICU	**0.10**
death	**0.31**
First Dose	**0.19**
Second Dose	**-0.15**
Booster	**-0.20**
Alpha	**0.71**
Beta	**0.34**
Delta	**-0.72**
Zeta	**0.26**
Theta	0.12
Gamma	**0.23**
Epsilon	**0.17**
Eta	**0.16**
Kappa	0.08
Iota	**0.15**
Mu	0.05
Lambda	0.06
GH490R	-0.07
Omicron	-0.07

Supplementary Table S 2: The contemporaneous bivariate correlations between each index and the weekly epidemiological variables for 2022 were analyzed across all countries. Significant results ($p < 0.05$) are highlighted in bold. Correlations were calculated by combining data from all available countries into a single vector for each variable.

	SI
New Cases	**0.73**
adm_Hosp	**0.41**
adm_ICU	**0.47**
death	**0.53**
First Dose	**0.50**
Second Dose	**0.44**
Booster	**0.44**
Delta	**0.54**
GH490R	**0.11**
Omicron	-0.06
Alpha	**0.13**
BA.2.75	**-0.27**
CH.1.1	**-0.14**
XBB	**-0.11**
XBB.1.5	-0.05

Supplementary Equation 1: complete fixed effects model.

$$
\begin{cases}
\mathbf{I}(t) = \beta_{01} + \beta_{11} \cdot I(t-1) + \beta_{21} \cdot NewCases(t-1) + \beta_{31} \cdot admHosp(t-1) + \beta_{41} \cdot admICU(t-1) \\
\quad +\beta_{51} \cdot death(t-1) + \beta_{61} \cdot FirstDose(t-1) + \beta_{71} \cdot SecondDose(t-1) + \beta_{81} \cdot BoosterDose(t-1) \\
\quad +\beta_{91} \cdot Alpha(t-1) + \beta_{101} \cdot Beta(t-1) + \beta_{111} \cdot Delta(t-1) + \beta_{121} \cdot Zeta(t-1), \\
\mathbf{nC}(t) = \beta_{02} + \beta_{12} \cdot I(t-1) + \beta_{22} \cdot NewCases(t-1) + \beta_{32} \cdot admHosp(t-1) + \beta_{42} \cdot admICU(t-1) \\
\quad +\beta_{52} \cdot death(t-1) + \beta_{62} \cdot FirstDose(t-1) + \beta_{72} \cdot SecondDose(t-1) + \beta_{82} \cdot BoosterDose(t-1) \\
\quad +\beta_{92} \cdot Alpha(t-1) + \beta_{102} \cdot Beta(t-1) + \beta_{112} \cdot Delta(t-1) + \beta_{122} \cdot Zeta(t-1), \\
\mathbf{aH}(t) = \beta_{03} + \beta_{13} \cdot I(t-1) + \beta_{23} \cdot NewCases(t-1) + \beta_{33} \cdot admHosp(t-1) + \beta_{43} \cdot admICU(t-1) \\
\quad +\beta_{53} \cdot death(t-1) + \beta_{63} \cdot FirstDose(t-1) + \beta_{73} \cdot SecondDose(t-1) + \beta_{83} \cdot BoosterDose(t-1) \\
\quad +\beta_{93} \cdot Alpha(t-1) + \beta_{103} \cdot Beta(t-1) + \beta_{113} \cdot Delta(t-1) + \beta_{123} \cdot Zeta(t-1), \\
\mathbf{aICU}(t) = \beta_{04} + \beta_{14} \cdot I(t-1) + \beta_{24} \cdot NewCases(t-1) + \beta_{34} \cdot admHosp(t-1) + \beta_{44} \cdot admICU(t-1) \\
\quad +\beta_{54} \cdot death(t-1) + \beta_{64} \cdot FirstDose(t-1) + \beta_{74} \cdot SecondDose(t-1) + \beta_{84} \cdot BoosterDose(t-1) \\
\quad +\beta_{94} \cdot Alpha(t-1) + \beta_{104} \cdot Beta(t-1) + \beta_{114} \cdot Delta(t-1) + \beta_{124} \cdot Zeta(t-1), \\
\mathbf{d}(t) = \beta_{05} + \beta_{15} \cdot I(t-1) + \beta_{25} \cdot NewCases(t-1) + \beta_{35} \cdot admHosp(t-1) + \beta_{45} \cdot admICU(t-1) \\
\quad +\beta_{55} \cdot death(t-1) + \beta_{65} \cdot FirstDose(t-1) + \beta_{75} \cdot SecondDose(t-1) + \beta_{85} \cdot BoosterDose(t-1) \\
\quad +\beta_{95} \cdot Alpha(t-1) + \beta_{105} \cdot Beta(t-1) + \beta_{115} \cdot Delta(t-1) + \beta_{125} \cdot Zeta(t-1), \\
\mathbf{fD}(t) = \beta_{06} + \beta_{16} \cdot I(t-1) + \beta_{26} \cdot NewCases(t-1) + \beta_{36} \cdot admHosp(t-1) + \beta_{46} \cdot admICU(t-1) \\
\quad +\beta_{56} \cdot death(t-1) + \beta_{66} \cdot FirstDose(t-1) + \beta_{76} \cdot SecondDose(t-1) + \beta_{86} \cdot BoosterDose(t-1) \\
\quad +\beta_{96} \cdot Alpha(t-1) + \beta_{106} \cdot Beta(t-1) + \beta_{116} \cdot Delta(t-1) + \beta_{126} \cdot Zeta(t-1), \\
\mathbf{sD}t) = \beta_{07} + \beta_{17} \cdot I(t-1) + \beta_{27} \cdot NewCases(t-1) + \beta_{37} \cdot admHosp(t-1) + \beta_{47} \cdot admICU(t-1) \\
\quad +\beta_{57} \cdot death(t-1) + \beta_{67} \cdot FirstDose(t-1) + \beta_{77} \cdot SecondDose(t-1) + \beta_{87} \cdot BoosterDose(t-1) \\
\quad +\beta_{97} \cdot Alpha(t-1) + \beta_{107} \cdot Beta(t-1) + \beta_{117} \cdot Delta(t-1) + \beta_{127} \cdot Zeta(t-1), \\
\mathbf{bD}(t) = \beta_{08} + \beta_{18} \cdot I(t-1) + \beta_{28} \cdot NewCases(t-1) + \beta_{38} \cdot admHosp(t-1) + \beta_{48} \cdot admICU(t-1) \\
\quad +\beta_{58} \cdot death(t-1) + \beta_{68} \cdot FirstDose(t-1) + \beta_{78} \cdot SecondDose(t-1) + \beta_{88} \cdot BoosterDose(t-1) \\
\quad +\beta_{98} \cdot Alpha(t-1) + \beta_{108} \cdot Beta(t-1) + \beta_{118} \cdot Delta(t-1) + \beta_{128} \cdot Zeta(t-1), \\
\mathbf{Al}(t) = \beta_{09} + \beta_{19} \cdot I(t-1) + \beta_{29} \cdot NewCases(t-1) + \beta_{39} \cdot admHosp(t-1) + \beta_{49} \cdot admICU(t-1) \\
\quad +\beta_{59} \cdot death(t-1) + \beta_{69} \cdot FirstDose(t-1) + \beta_{79} \cdot SecondDose(t-1) + \beta_{89} \cdot BoosterDose(t-1) \\
\quad +\beta_{99} \cdot Alpha(t-1) + \beta_{109} \cdot Beta(t-1) + \beta_{119} \cdot Delta(t-1) + \beta_{129} \cdot Zeta(t-1), \\
\mathbf{Be}(t) = \beta_{010} + \beta_{110} \cdot I(t-1) + \beta_{210} \cdot NewCases(t-1) + \beta_{310} \cdot admHosp(t-1) + \beta_{410} \cdot admICU(t-1) \\
\quad +\beta_{510} \cdot death(t-1) + \beta_{610} \cdot FirstDose(t-1) + \beta_{710} \cdot SecondDose(t-1) + \beta_{810} \cdot BoosterDose(t-1) \\
\quad +\beta_{910} \cdot Alpha(t-1) + \beta_{1010} \cdot Beta(t-1) + \beta_{1110} \cdot Delta(t-1) + \beta_{1210} \cdot Zeta(t-1), \\
\mathbf{De}(t) = \beta_{011} + \beta_{111} \cdot I(t-1) + \beta_{211} \cdot NewCases(t-1) + \beta_{311} \cdot admHosp(t-1) + \beta_{411} \cdot admICU(t-1) \\
\quad +\beta_{511} \cdot death(t-1) + \beta_{611} \cdot FirstDose(t-1) + \beta_{711} \cdot SecondDose(t-1) + \beta_{811} \cdot BoosterDose(t-1) \\
\quad +\beta_{911} \cdot Alpha(t-1) + \beta_{1011} \cdot Beta(t-1) + \beta_{1111} \cdot Delta(t-1) + \beta_{1211} \cdot Zeta(t-1), \\
\mathbf{Ze}(t) = \beta_{012} + \beta_{112} \cdot I(t-1) + \beta_{212} \cdot NewCases(t-1) + \beta_{312} \cdot admHosp(t-1) + \beta_{412} \cdot admICU(t-1) \\
\quad +\beta_{512} \cdot death(t-1) + \beta_{612} \cdot FirstDose(t-1) + \beta_{712} \cdot SecondDose(t-1) + \beta_{812} \cdot BoosterDose(t-1) \\
\quad +\beta_{912} \cdot Alpha(t-1) + \beta_{1012} \cdot Beta(t-1) + \beta_{1112} \cdot Delta(t-1) + \beta_{1212} \cdot Zeta(t-1).
\end{cases}
\tag{1}
$$
